# Dissecting the mechanisms responsible for the multiple insecticide resistance phenotype in *Anopheles gambiae* s.s., M form, from Vallée du Kou, Burkina Faso

**DOI:** 10.1016/j.gene.2013.01.036

**Published:** 2013-04-25

**Authors:** Rachel M. Kwiatkowska, Naomi Platt, Rodolphe Poupardin, Helen Irving, Roch K. Dabire, Sara Mitchell, Christopher M. Jones, Abdoulaye Diabaté, Hilary Ranson, Charles S. Wondji

**Affiliations:** a Liverpool School of Tropical Medicine, Vector Biology Department, UK; b IRSS/Centre Muraz, BP 390 Bobo-Dioulasso, Burkina Faso

**Keywords:** VK, Vallée du Kou, DDT, dichlorodiphenyltrichloroethane, *kdr*, knockdown resistance, RDL, resistance to dieldrin, GO, Gene Ontology, MoH, Ministry of Health, IRS, indoor residual spraying, LLINs, long lasting insecticide nets, *AChE*, acetylcholinesterase, GSTs, glutathione-S-transferases, WHO, World Health Organization, SINE, short interspersed element, FC, fold change, *sHSP*, small Heat Shock Protein, qRT-PCR, quantitative reverse transcriptase polymerase chain reaction, GABA, gamma-aminobutyric acid, *Anopheles gambiae*, Malaria, Insecticide resistance, Burkina Faso, Microarray

## Abstract

With the exception of target site mutations, insecticide resistance mechanisms in the principle malaria vector *Anopheles gambiae*, remains largely uncharacterized in Burkina Faso.

Here we detected high prevalence of resistance in Vallée du Kou (VK) to pyrethroids, DDT and dieldrin, moderate level for carbamates and full susceptibility to organophosphates. High frequencies of L1014F *kdr* (75%) and *Rdl* (87%) mutations were observed showing strong correlation with pyrethroids/DDT and dieldrin resistance. The frequency of *ace1^R^* mutation was low even in carbamate resistant mosquitoes. Microarray analysis identified genes significantly over-transcribed in VK. These include the cytochrome P450 genes, *CYP6P3* and *CYP6Z2*, previously associated with pyrethroid resistance. Gene Ontology (GO) enrichment analysis suggested that elevated neurotransmitter activity is associated with resistance, with the over-transcription of target site resistance genes such as acetylcholinesterase and the GABA receptor. A rhodopsin receptor gene previously associated with pyrethroid resistance in *Culex pipiens pallens* was also over-transcribed in VK.

This study highlights the complex network of mechanisms conferring multiple resistance in malaria vectors and such information should be taken into account when designing and implementing resistance control strategies.

## Highlights

► High pyrethroids, DDT and dieldrin resistance in VK, moderate for carbamate ► High frequencies *kdr* and *Rdl* mutations in VK with strong correlation with resistance ► Detection using microarray of genes upregulated in VK notably *CYP6P3* and *CYP6Z2* P450s ► Neurotransmitter activity GO terms enriched in VK; *Ace-1*/GABA receptor upregulated ► A rhodopsin gene associated with resistance in *Culex p. pallens* upregulated in VK

## 1 Introduction

Malaria is endemic in Burkina Faso (West Africa) where it is the main cause of morbidity and mortality (MoH, Burkina Faso). Current efforts to control malaria in Burkina Faso, as elsewhere in Africa, rely on vector control methods such as long lasting insecticides nets (LLINs) and indoor residual spraying (IRS) (Dabiré et al., 2012).

Insecticide resistance in malaria vectors is widely reported in Burkina Faso and has been linked to the heavy agricultural use of insecticides (Dabiré et al., 2012; Diabate et al., 2002a). One of the locations highly impacted by agricultural selection pressure is the region of Vallée du Kou (VK) in the south west of the country, an area comprising seven villages. Vallée du Kou is surrounded by agricultural land which has long been exposed to pesticides, contributing to the selection of resistance in malaria vectors (Diabate et al., 2002a). For this reason, Vallée du Kou has regularly been used to monitor patterns of insecticide resistance in malaria vectors, most notably *Anopheles gambiae* (Dabire et al., 2008).

The two major causes of insecticide resistance in malaria vectors are alterations in the target sites and increase in the rate of insecticide metabolism. One of the main target site mutations is the ‘knock-down resistance’ mutation (*kdr*) conferring resistance to pyrethroids and DDT. In *A.. gambiae*, two amino acid changes in the voltage-gated sodium channel gene at codon 1014, a leucine to phenylalanine substitution also known as 1014F (Martinez-Torres et al., 1998) and a leucine to serine substitution also known as 1014S (Ranson et al., 2000), are involved. A single amino acid substitution of glycine to serine at position 119 in the catalytic domain of the acetylcholinesterase (*AChE*) gene confers resistance to both organophosphates and carbamates in *A. gambiae* (Weill et al., 2004). Resistance to cyclodienes such as dieldrin is conferred by either a change of alanine to serine or to glycine in the GABA receptor gene at codon 296 (Du et al., 2005). Metabolic resistance is caused primarily by three enzyme families, the cytochrome P450s, the esterases and glutathione-S-transferases (GSTs)(Hemingway and Ranson, 2000).

Since the first report of resistance to insecticide in this VK *A. gambiae* population (Chandre et al., 1999), a constant increase in the level of pyrethroid and DDT resistance has been observed, associated with an increase in the frequency of the 1014F *kdr* allele (Dabire et al., 2008; Diabate et al., 2002b). In addition to this *kdr* mutation, the G119S mutation in the acetylcholinesterase gene (*Ace-1^R^*), known to confer resistance to organophosphates and carbamates, has been detected in the *A. gambiae* population of VK in 2005, but at low frequencies (0.03 M form, 0.37 S form) (Dabire et al., 2008). Other resistance mechanisms, such as metabolic resistance, undoubtedly play an important role in the insecticide resistance observed in field populations of malaria vectors across Africa (Djouaka et al., 2008; Muller et al., 2008a), but the potential role of these mechanisms, and the genes underlying the resistance trait, have not been widely explored in mosquitoes from Burkina Faso. Additionally, insecticide resistance profiling in VK have mainly focused on two of the seven villages: VK5 (Diabate et al., 2002b) and VK7 (Dabire et al., 2008; Diabate et al., 2002b). A broad resistance pattern from the seven villages has not yet been established.

This study aims to build on previous studies from Vallée du Kou to establish the extent and distribution of insecticide resistance in populations of *A. gambiae* from all seven villages against the four major classes of insecticide. The underlying resistance mechanisms are dissected by assessing the correlation between resistance phenotypes and target-site mutations and by investigating the contribution of metabolic resistance using a microarray approach.

## 2 Results

### 2.1 Mosquito collections and bioassays

All bioassay tests were carried out using F_1_ mosquitoes generated from indoor-collected blood-fed female *A. gambiae s.l.* from VK1 (n = 226), VK2 (= 241), VK3 (n = 292), VK6 (n = 311) and VK7 (n = 225). Only few mosquitoes were collected in VK4 and VK5 due to low water levels in the rice paddies in these two villages. In total, 2302 mosquitoes from the five villages were exposed to the diagnostic concentration of insecticide defined by WHO (WHO, 1998).

**Fig. 1:**
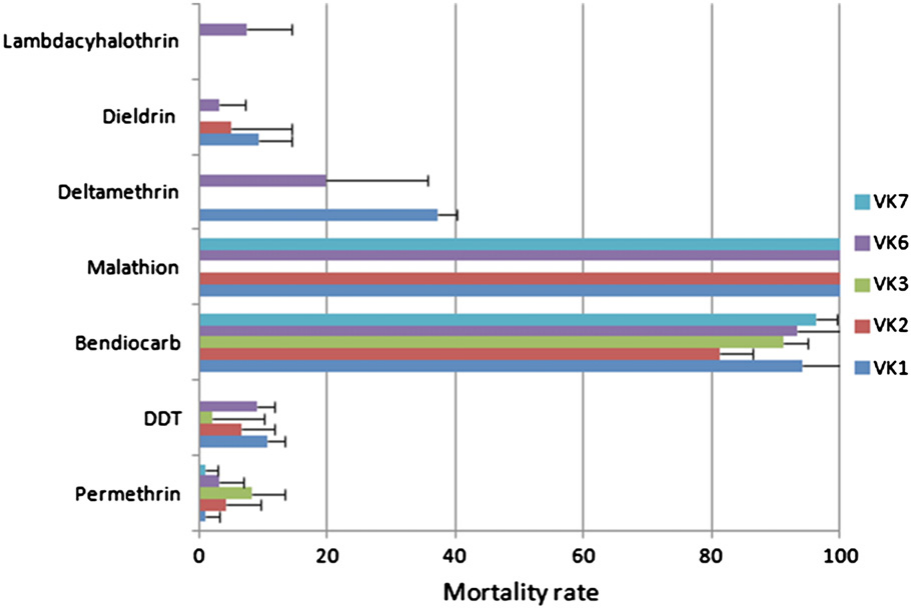
Susceptibility/resistance status of the *A. gambiae* M form population of Vallée du Kou to the main insecticides. Due to sample size limitations, not all insecticides were tested in all villages. Absence of bars for some villages indicates that there is no data for the tested insecticide.

Pooling the data from all five villages, there is a high prevalence of resistance to permethrin (3.4% mortality), DDT (10% mortality), dieldrin (5.9% mortality), deltamethrin (24.8% mortality), and lambda-cyhalothrin (7.3% mortality). Malathion was the only insecticide found to be fully effective (100% mortality). A small number of mosquitoes survived bendiocarb exposure (91.1% mortality) (Fig. 1; Table S1). Bioassay results were fairly uniform between villages (Fig. 1; Table S1) except in the case of bendiocarb exposure, where mortality was significantly lower in VK2 than in other villages (81.2% mortality compared with mean 92.6% from other villages. *χ*^2^ = 18.9, P = 0.000).

### 2.2 Genotyping of target-site mutations

The SINE PCR carried out on 15 field-collected females from each village and on 50 F_1_ samples indicated that they were all *A. gambiae* s.s. from the M molecular form.

#### 2.2.1 *kdr* mutation

The pyrosequencing method unambiguously scored the three genotypes of the L1014F *kdr* (Fig. S1) and also genotyped the L1014S *kdr* T/C position simultaneously. The frequency of the 1014F *kdr* allele was 75% in the 100 F_0_ females tested. No 1014S alleles were detected. Overall the T/T resistant homozygote genotype was present at 53% in the field collected F_0_ females, the heterozygote A/T present at 43% while the homozygote susceptible genotype A/A was observed at only 4%.

The frequencies of the *kdr* genotypes significantly differed between susceptible and resistant phenotypes for both permethrin and DDT (permethrin, *χ*^2^ = 101, df = 2 and P = 0.000; DDT, *χ*^2^ = 64.9, df = 2 and P = 0.000) (Table S2). A significant association was observed between the *kdr* mutation and resistance to permethrin and DDT with odds ratio of 2.7 (P < 0.05) and 2.8 (P < 0.05) respectively when comparing the allele counts between phenotypes.

**Fig. 2:**
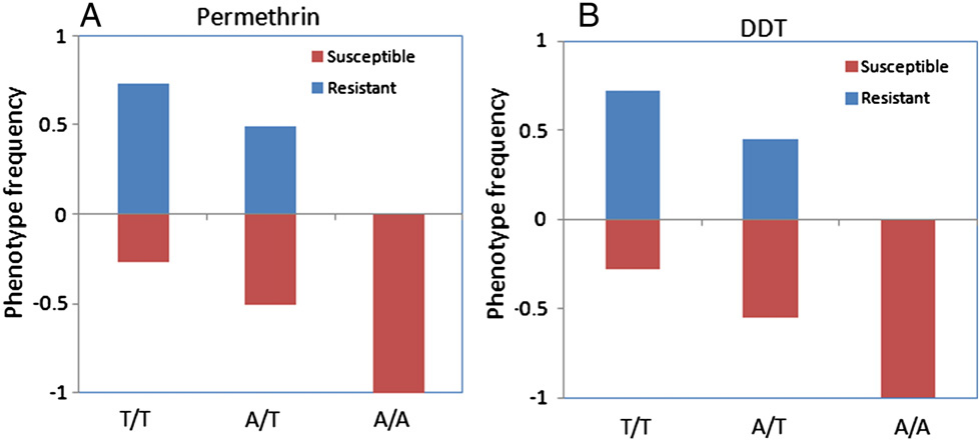
Correlation between resistance phenotype and the L1014F genotypes; A) is for permethrin and B) for DDT. The frequency of each genotype is plotted in each phenotype to indicate differences in survival between the genotypes (T/T: resistant L1014F *kdr* genotype; A/T: heterozygote; A/A: wild type susceptible).

In both permethrin and DDT exposed samples, the majority of mosquitoes with the T/T homozygote *kdr* genotype were resistant (73.1 and 72.0% respectively for permethrin and DDT) while approximately half of the heterozygote mosquitoes were resistant (49.2% for permethrin and 45.7% for DDT), and none of the wild-type (susceptible) genotype were resistant (Figs. 2A and B). The frequencies of the A/T heterozygote genotype in resistant and susceptible DDT phenotypes (52% and 62% respectively) were significantly higher compared to the permethrin exposed mosquitoes (41% and 43% respectively) (*χ*^2^ = 7.75, P = 0.005).

#### 2.2.2 *AChE* mutation

The pyrosequencing method successfully scored the A/G mutation according to the expected nucleotide peaks on the histograms (Fig. S2). The 119S *ace1^R^* allele was present at a low frequency of 4.2% (n = 124) and always as heterozygote. Thirty-five bendiocarb survivors and 24 females dead after exposure were also genotyped for *ace1^R^*. Only four individuals contained the *ace1^R^* allele and all of these were bendiocarb survivors, suggesting a correlation between the *ace1^R^* allele and resistance to bendiocarb (Table S3). The overwhelming predominance of the susceptible *Ace1^S^* genotype, even in resistant mosquitoes, is a strong indicator that other resistance mechanisms are mainly responsible for the observed carbamate resistance.

#### 2.2.3 Resistance to dieldrin (*Rdl*) mutation

**Fig. 3:**
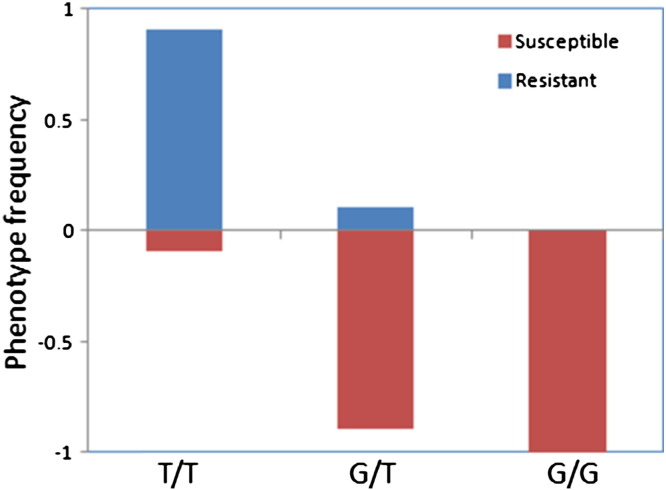
Correlation between resistance phenotype to dieldrin and the A296S genotypes. The frequency of each genotype is plotted in each phenotype to indicate differences in survival between the genotypes (T/T: resistant *Rdl* genotype; G/T: heterozygote; G/G: wild type susceptible).

The A296S *Rdl* mutation, unambiguously scored by the pyrosequencing method (Fig. S3), was present at a high frequency in VK (87%) (n = 94). Overall, the T/T resistant homozygote genotype was present in F_0_ females at 77.7%, the heterozygote G/T present at 19.1% while the homozygote susceptible genotype G/G was observed at only 3.2%. A subset of 76 dieldrin phenotyped individuals was also scored for the *Rdl* mutation. Resistant samples were almost exclusively homozygous for the *Rdl* mutation (94%). In contrast, over half of the susceptible mosquitoes were heterozygous (G/T 60%), 30% wild type (G/G) and only 10% homozygous T/T (Table S4). The difference between the phenotypic composition of each *RDL* genotype is highly significant (*χ*^2^ = 257, P = 0.00). A significant association was observed between the RDL mutation and resistance to dieldrin with an odds ratio of 39.4 (P < 0.0001) when comparing the allele counts between phenotypes. Homozygotes were almost all resistant to dieldrin (90.9%R) (Fig. 3), whereas heterozygotes were for the most part susceptible (89.2%S) indicating that this mutation may be recessive. The wild type genotypes were all from the susceptible subset.

### 2.3 Microarray analysis

Microarrays were used to compare the genome-wide transcriptome between VK6 samples and the susceptible Ngoussou strain (also M form). The quality control (QC) analysis of all the samples after normalization indicated that 5576 out of 14,999 probes or entities (37.2%) passed the filtering based on flags present or marginal in at least 1 out of the 5 samples used in this experiment. Using this 5576 probes set, the number of differentially expressed probes (≥ 2-fold) between the VK and Ngoussou samples is 1493, 1075, 786 and 108 respectively for P values of 0.05, 0.01, 0.005 and 0.001 (Fig. S4). For subsequent analysis, a P value of 0.005 was selected which resulted in a subset of 429 probes over-transcribed in the resistant VK sample and 357 probes under-transcribed compared to the susceptible Ngoussou strain. The top fifty probes in the over-transcribed subset, based on fold-change are listed in Table 1 while the top 30 probes the most under-transcribed are listed in Table 2. A complete list of the 786 probes is provided in the supplementary files (Table S5).

#### 2.3.1 Over-transcribed genes in VK

The three probes with the highest fold change (FC > 74) in VK relative to Ngoussou belong to three different transcripts (AGAP007160-RA, RB and RC) encoding a small Heat-Shock Protein *sHSP20* (168 amino acids) on chromosome 2L. A putative proteinase inhibitor (AGAP002878-RA) on chromosome 2R also appears to be highly over-transcribed (FC 73.1) as does a mitochondrial gene AGAP006879 (FC 26.4) (chromosome 2L) which encodes the subunit E of the ATP synthase involved in transmembrane ion transport.

In addition, a putative rhodopsin receptor gene *GPROP3* (AGAP012982), which is an ortholog of the *NYD-OP7* gene associated with deltamethrin resistance in the mosquito *Culex pipiens pallens* (Hu et al., 2007), is also over-transcribed in VK (FC 16.6) besides two other genes known to interact with rhodopsin; arrestin (AGAP006263) and rhodopsin receptor 1 (AGAP013149).

In total, twenty-one probes, representing 11 genes with putative detoxification function or previously associated with insecticide resistance were over-transcribed (Table S6). Most notable were cytochrome P450s with six genes over-transcribed in VK. *CYZ6Z2* and *CYP6P3* were the most over-transcribed of this gene family and consistently for their three probes (FC 20.5, 11.1 and 7 for *CYP6Z2* and 10, 7.8 and 7.4 for *CYP6P3*). In addition, *CYP6AA1* (all 3 probes; FC 2.4), *CYP6AG2* (FC 2.3), *CYP6M3* (FC 2.0) and *CYP6P1* (FC 2.0) were also over-transcribed. Other over-transcribed detoxification genes include an aldehyde oxidase (FC 16.5) previously associated with insecticide resistance in *Culex quinquefasciatus* (Giraudo et al., 2011), two peroxidase genes *PX13A* (FC 3.5) and *GPX3* (FC 2.6) and a glutathione-S-transferase gene *GSTZ1* (FC 2.6). Surprisingly, 3 probes of the acetylcholinesterase gene *Ace-1*, were all over-transcribed in VK (FC 2.5) as well as a probe for *Ace2* the second acetylcholinesterase gene in *A. gambiae* (FC 2.7). The *Ace-1* gene is normally associated with a target-site mutation conferring carbamate/organophosphate resistance. The GABA receptor gene (AGAP006028) associated with target site resistance to dieldrin was also over-transcribed in VK (FC 5.5).

Several other genes with diverse functions are also over-transcribed in VK. Among these is a group of genes involved in peptidase regulation including a chymotrypsin (AGAP009828) (FC 10.4) and a serine protease (AGAP009212) (FC9.8). Other gene categories include salivary protein genes (AGAP006504 (FC 15.5), AGAP003444 (FC 11.9)), cuticular protein genes (CPR21 (AGAP005996) (FC 12.4)) and an UDP glucosyl glucuronosyl transferase gene (AGAP007990) (FC 7.7).

#### 2.3.2 Under-transcribed genes

No specific gene family is predominant among the under-transcribed genes in VK compared to the susceptible Ngoussou strain and most probes in this list are from genes with unknown functions. However, several GSTs were down regulated (Table S7) including *GST1-5* (FC 3.2) *GSTE3* (FC 2.6), *GSTE4* (FC 2.5), and *GST1-6* (FC 2.1) and *GSTU1* (FC 2.4). Overall, 35 probes from detoxification genes were under-transcribed in VK including one cytochrome P450, *CYP6AK1* (FC 3.3).

#### 2.3.3 Functional profiling of over-transcribed genes using GO enrichment analysis

GO enrichment analysis with both DAVID functional and Blast2Go produced similar results. This analysis confirms that activities related to neuro-muscular and neurotransmitter activities were the most enriched in VK with enrichment scores of 2.36 and 2.04 respectively recorded for these groups (Table 3). GO terms associated with neurotransmitter activity include for the cellular component category terms such as presynaptic membrane, postsynaptic membrane, the terminal buttons, the neuromuscular junction and synaptic vesicles term (or neurotransmitter vesicles) (Table S8). For the molecular function category, it includes terms such as neurotransmitter transporter activity, 4-aminobutyrate transaminase activity, acetylcholinesterase activity and cholinesterase activity (Table S8) in line with the over-expression of the GABA receptor or the *Ace-1* and *Ace-2* genes observed in this study. GO terms associated with neuro-muscular activity include, for the cellular component category, terms such as A band, Z disk and troponin complex, apical cortex, basolateral plasma membrane and vacuolar proton-transporting V-type ATPase, V1 domain (Table S8). These terms are associated with the muscular system of insects related to cell membrane activities linked with muscular contraction. For the molecular function category, it includes terms such as actin binding, tropomyosin binding and protein N-acetylglucosaminyltransferase activity (Table S8), an enzyme which belongs to the family of glycosyltransferases notably over-transcribed in VK.

An enrichment of stress response activity was also observed (with a score of 2.06) as a response to heat or temperature stimulus. This enrichment of stress response activity is in line with the highest over-expression seen in VK for heat shock protein AGAP007160. The DAVID functional analysis which takes into account other parameters such as the protein ID using INTERPRO or the SMART ID (Huang da et al., 2009) also revealed, contrary to Blast2Go, an enrichment of detoxification activity through cytochrome P450 genes but also the aldehyde oxidase gene in correlation with the over-expression of some P450s such as *CYP6P3* and *CYP6Z2* and an aldehyde oxidase in VK.

### 2.4 Validation of the microarray up-regulation with qRT-PCR

**Fig. 4:**
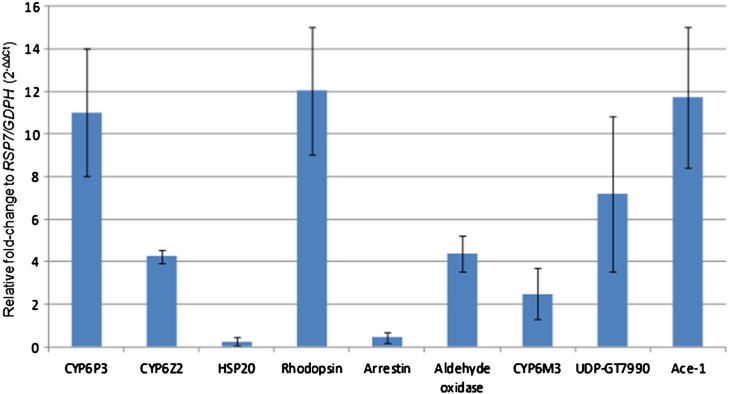
qRT-PCR expression profile of the six candidate genes in females of the resistant VK population and the susceptible strain Ngoussou. The relative fold change of the 2^− ΔΔCt^ of each gene between VK and Ngoussou are represented on the Y axis.

Nine of the most over-transcribed genes in VK, the heat shock protein *sHSP20* (AGAP007160), *CYP6Z2*, *CYP6P3*, *CYP6M3*, aldehyde oxidase (AGAP006226), an UDP glucosyl-transferase (AGAP007990), acetylcholinesterase 1 (Ace-1), the putative rhodopsin receptor AGAP012982 and arrestin (AGAP006263) were selected for validation by qPCR. All primer pairs tested had amplification efficiencies between 90 and 110%. A significant over-expression in VK was confirmed for seven genes when their (2^− ΔΔCt^) relative expression was compared between VK and Ngoussou after normalization with the two housekeeping genes *RSP7* and GDPH (Fig. 4). The highest fold-change is observed for the rhodopsin gene with a 12.1-fold up-regulation in VK compared to the susceptible Ngoussou sample (FC 16.6 for microarray). The P450 *CYP6P3* is 11.0-fold over-transcribed in VK (Average FC 8.5 for microarray) while *CYP6Z2* is also over-transcribed in this resistant sample at 4.5-fold (average FC 12.7 for microarray), which is similar to the up-regulation observed for the aldehyde oxidase gene (4.4-fold change) (FC 16.5 for microarray). Similarly, the qPCR profiles of *CYP6M3*, acetylcholinesterase (Ace-1) and the UDP glucosyl-transferase gene correlated with their microarray results showing an over-transcription in VK. No over-transcription was observed for the heat shock protein *sHSP20* (AGAP007160) and for the Arrestin gene with both being rather under-transcribed in VK from this qRT-PCR result contrary to the microarray results.

## 3 Discussion

This study has provided an update on the current levels of resistance and the underlying resistance mechanisms in *A. gambiae* s.s., M form, in the Vallée du Kou region of Burkina Faso.

The WHO bioassay results indicated a high prevalence of resistance to pyrethroids in VK villages for both Type I pyrethroid (permethrin) or Type II (deltamethrin and lambda-cyhalothrin). This pattern is broadly similar between the five villages indicating an extensive gene flow between these populations (potentially constituting a single panmictic population) or similar selection pressure. The proportion of *A. gambiae* surviving permethrin exposure has increased considerably since 1999 (61.6% mortality in 1999 (Chandre et al., 1999) vs. 3.4% in 2010 reported here). Overall, mortality rates are lower for permethrin (3.4%) than for deltamethrin (24.8%) similar to trends usually observed in *A. gambiae* (Ramphul et al., 2009; Ranson et al., 2009). A recent nationwide susceptibility study of *A. gambiae* populations across Burkina Faso revealed that mortality rates for permethrin ranged from 20% in Batié to 97% in Manga (Dabiré et al., 2012). With only 3.4% mortality, VK populations exhibit the highest prevalence of resistance to permethrin in the country. Furthermore, the high levels of resistance to deltamethrin are of real concern given the widespread distribution of LLINs impregnated with this insecticide as part of the National Malaria Control Programme. The efficacy of these LLINs could be negatively affected by this resistance as previously observed in Benin (N'Guessan et al., 2007). A high prevalence of resistance to DDT with mortality rates ranging from 2 to 10.5% between the VK villages was observed, consistent with previous report in VK in 1999 and 2005 (Chandre et al., 1999; Dabire et al., 2008).

The high frequency of L1014F *kdr* mutation (75%) in this VK population confirms the trend of an increase of this mutation in VK as previously reported. Indeed, the 1014F *kdr* allele, only detected at a very low frequency of 2% in 2000 (Diabate et al., 2002b) and 20% in 2005 (Dabire et al., 2008) had risen to 75% by 2010. This continued rise of this *kdr* frequency is an indication that this M form of VK is still undergoing selection for resistance either through the agricultural use of insecticides or the widespread use of LLINs in VK (Dabire et al., 2008; Diabate et al., 2002a). Analysis of the association between *kdr* genotypes and resistance indicated that the L1014F mutation was associated with both permethrin and DDT resistance as reported previously (Donnelly et al., 2009).

Overall a co-dominance like-effect was observed for the L1014F mutation for both permethrin and DDT as the heterozygote individuals exhibited intermediate resistance levels contrary to the recessive effect originally described for this mutation (Martinez-Torres et al., 1998). A similar analysis of a population of *A. gambiae* from Uganda found a similar co-dominance effect for L1014S and DDT but not for permethrin resistance (Ramphul et al., 2009). The distribution of the L1014S mutation in *A. gambiae* s.s. has expanded from its origin in East Africa (Ranson et al., 2000) to Central (Reimer et al., 2008) and West Africa (Djegbe et al., 2011), however, it was not found from M form *A. gambiae* in VK in this study.

Resistance to the cyclodiene dieldrin was also prevalent in all VK populations with mortality ranging from 3.1 to 9.2% indicating that resistance against this insecticide, already reported since the 1960–70s in Burkina Faso (Hamon et al., 1968), remains established despite the fact that it is no longer used in public health. A similar situation was also recently observed for *Anopheles funestus* in Burkina Faso (Wondji et al., 2011). Because a fitness cost has been shown to be associated with dieldrin resistance (Rowland, 1991a, 1991b) it is rather expected that in the absence of dieldrin selection, this resistance will revert to susceptibility as previously observed in Nigeria (Hamon and Garrett-Jones, 1963). Therefore, the persistence of dieldrin resistance in *A. gambiae* in VK may be the result of the use of agrochemicals targeting the GABA receptor in the agricultural sector, rather than reflecting a lack of fitness cost as observed in *C. pipiens* and *Aedes albopictus* populations in La Reunion (Tantely et al., 2010). Beside the *RDL* mutation, some of the genes over-transcribed could be associated to dieldrin resistance. This includes the up-regulation of the GABA receptor gene and the enrichment of the 4-aminobutyrate transaminase activity term, an enzyme which catalyzes the conversion of the 4-aminobutanoic acid (GABA) and 2-oxoglutarate into succinic semialdehyde and glutamate.

The organophosphate malathion remained 100% effective in this population indicating that organophosphates could be considered as an alternative insecticide for IRS campaign around VK. The prevalence of bendiocarb resistance also remains low. However, the presence of *ace-1* mutations in the population suggests that resistance to these insecticide classes could increase rapidly. There is no obvious explanation for the higher bendiocarb resistance observed in VK2 as this village directly borders VK1 and both are surrounded by rice paddies. It will be necessary to monitor VK2 further to elucidate the likely reason of this difference. The low *ace1^R^* frequency in the resistant bendiocarb mosquitoes indicates that the *G119S* mutation may not have a major role in the carbamate resistance in this population. Metabolic resistance is probably the cause of this resistance. The enrichment of GO terms associated with acetylcholinesterase activity and cholinesterase activity in this study indicates that besides the *Ace-1* G119S mutation, the over-expression of the two acetylcholinesterase genes is possibly associated with the bendiocarb resistance. This over-expression of *Ace-1* could also be associated with the presence of a duplicated copy of this gene in some resistant field populations of *A. gambiae* (Djogbenou et al., 2009).

The contribution of the metabolic resistance mechanisms to the multiple resistance patterns of the VK population is supported by the microarray results. This contribution is shown through the up-regulation of genes involved in insecticide detoxifications such as P450 genes but also by the over-representation of GO terms from activities associated with resistance in this population. The detection of the detoxification genes such as the P450 *CYP6P3* previously shown to confer pyrethroid resistance in other populations of *A. gambiae* (Djouaka et al., 2008; Muller et al., 2008a) suggests that metabolic resistance is also contributing to the high level of pyrethroid resistance in VK. The up-regulation of other P450 genes such as *CYP6Z*2 and *CYP6AA1*, found over-transcribed in pyrethroid resistant strain (David et al., 2005) further supports this role. Among other genes with a potential role in pyrethroid resistance are the peroxidases *PX13A* and *GPX3*, which are also over-transcribed in VK. Peroxidases and glutathione peroxidases are known to reduce the damaging effects of reactive oxygen species released by insecticides (Vontas et al., 2005) and are also associated with pyrethroid resistance (Muller et al., 2008b). Glucuronosyl transferase genes are responsible for the process of glucuronidation, which plays a major part in phase II metabolism of xenobiotics. Glucuronidation represents a major pathway which enhances the elimination of many lipophilic xenobiotics and endobiotics to more water-soluble compounds (King et al., 2000). Therefore, the over-expression of genes from this family could feasibly play a role in insecticide resistance in this population. However, the difference in the breeding environments between the VK field strain and the Ngoussou lab strain could also explain this expression pattern. Unfortunately due to high resistance in VK no suitable field susceptible sample with the same genetic background could be found to compare with the resistant VK samples.

Apart from the known detoxification genes, the over-transcription of the *GPROP3* rhodopsin receptor gene (AGAP012982-RA) in VK, from both microarray and qPCR analyses, is an interesting observation as a gene from this family (made of 11 genes in *A. gambiae*), *NYD-OP7* with 86% identities to GPR0P3, has previously been associated with deltamethrin resistance in another mosquito species *C. pipiens pallens* (Hu et al., 2007). *NYD-OP7* was shown to independently confer deltamethrin resistance with expression of this gene in the mosquito C6/36 cell line conferring moderate deltamethrin resistance. The up-regulation of the rhodopsin gene in VK is further evidence that increased expression of an opsin gene may have a role in pyrethroid resistance. Additionally, it has been observed that *Drosophila* UV-sensitive opsins (*Rh3* and *Rh4*) and blue-sensitive opsin (*NinaE*, ortholog of *GPR0P3*) were over-transcribed in *Drosophila* DDT-resistant strain (Pedra et al., 2004), suggesting that *opsins* gene may also contribute to increased tolerance to DDT.

The highest expression of the heat-shock protein *sHSP20* was not confirmed by qRT-PCR. This should be further investigated to establish the reason of this discrepancy. However, this could be due to sequence conservation between members of this gene family inducing non-specific hybridization of the qRT-PCR primers used in this study.

On top of the identification of candidate genes associated with the multiple resistances in the VK population, the analysis of the whole transcriptome of *A. gambiae* in this study has allowed to establish the broader picture of insecticide resistance mechanism. The higher expression of neurotransmitter genes associated with target site resistance such as acetylcholinesterase and the GABA receptor gene indicates that target site resistance may also be associated to the over-expression of target-site resistance genes. The reason of such over-expression remains unclear, however, we could hypothesize that it may provide extra molecules/receptors to compensate for those which may be compromised by insecticides or this may reflect gene amplification events through duplication as seen in *Ace-1*. This will need to be further investigated.

## 4 Conclusion

This investigation into the insecticide resistance pattern in Vallée du Kou, Burkina Faso, has established the extent and investigated the causes of the observed multiple resistance. Insecticide resistance in the major malaria vector *A. gambiae*, M form, in VK is extremely high to all but the organophosphate and carbamate insecticide classes. We have confirmed the importance of target site resistance but also highlighted the complexities underlying insecticide resistance in malaria vectors, with resistance phenotypes conferred by multiple molecular mechanisms. This complexity should be taken into consideration by vector control programs when designing and implementing insecticide resistance control strategies.

## 5 Methods

### 5.1 Field collection

Samples were collected from Vallée du Kou (VK), in South-West Burkina Faso. The region spans 1200 ha from 4° 24′ 42″ longitude west to 11° 23′ 14″ latitude north. Vallée du Kou is an area of wooded savannah consisting of seven adjoining villages (Fig. S5) referred as VK1 to VK7. Semi-permanent irrigation systems are in place dating back to 1972 (Dabire et al., 2008) for the cultivation of rice and in addition the villages are permanently supplied by the river Kou. Mean annual rainfall is estimated at 1200 mm per annum and rice is the major crop. Rice paddies form ideal mosquito breeding sites, as do the depressions and ponds that form in the dirt roads through the villages. The savannah region surrounding Vallée du Kou is largely used for cotton and vegetable cultivation, and has seen decades of heavy insecticide exposure (Dabire et al., 2008).

Indoor resting collections of blood fed and gravid female *Anopheles* mosquitoes were conducted between the hours of 08:00 am and 12:00 pm in mid- to late April 2010 using manual aspirators. Mosquitoes were transported to the insectaries of the Centre Muraz Institute, Bobo Dioulasso, located 30 km south of VK, and were allowed to lay eggs in cages. After oviposition, these females were stored in Eppendorf tubes with silica gel for further characterization. Emerging larvae were then reared to adults and transferred into cages for use in bioassays.

### 5.2 Bioassays

Insecticide susceptibility assays were carried out using 2–5 day-old F_1_ female adults following WHO protocol (WHO, 1998). Approximately 20–25 mosquitoes per tube were exposed to insecticide-impregnated filter papers, supplied by WHO, for 1 h before being transferred to a clean holding tube supplied with 10% sugar. Mortality was then determined 24 h later. Seven compounds representative of the four major insecticide classes were tested: the pyrethroids permethrin (0.75%), deltamethrin (0.05%) and lambda-cyhalothrin (0.05%); the carbamate bendiocarb (0.01%); the organophosphate malathion (5%) and the organochlorines DDT (4%) and dieldrin (4%). After the 24 hour recovery period, surviving mosquitoes were immediately stored in Eppendorf tubes containing RNAlater solution (Ambion) for preservation, while dead females were stored in silica gel tubes.

### 5.3 Species and molecular form identification

Genomic DNA was extracted from legs and wings of indoor collected *A. gambiae s.l*. females using the LIVAK technique (Livak, 1984). Species ID of these specimens and the molecular form of *A. gambiae* s.s. specimens was identified using the SINE PCR protocol (Santolamazza et al., 2008).

### 5.4 Genotyping of target site mutations using the pyrosequencing method

Both the L1014F and L1014S *kdr* mutations were genotyped using the pyrosequencing method in resistant (n = 24) and susceptible (n = 14, as few susceptible were available due to high resistance prevalence) permethrin mosquitoes and DDT resistant (n = 23) and susceptible (n = 21) mosquitoes in order to assess the correlation between *kdr* mutations and resistance phenotype. The same was done for the G119S *AChE* mutation, conferring carbamate/organophosphate resistance (35 resistant and 24 susceptible mosquitoes) and also for the A296S *RDL* mutation conferring dieldrin resistance (36 resistant and 40 susceptible mosquitoes) by genotyping a set of resistant and susceptible mosquitoes to bendiocarb and dieldrin respectively. Association between resistance phenotypes and the genotypes of the resistance mutation was assessed by estimating the odds ratios and the statistical significance based on the Fisher's exact probability test. Additionally, the frequency of the *kdr*, *Ace-1* and *RDL* mutations in the population was assessed by genotyping 100 field-collected female mosquitoes.

Pyrosequencing reactions were carried out as described previously (Wondji et al., 2007) and primer sequences are given in Table S9.

### 5.5 Microarray

The 8 × 15 K Agilent microarray design chip (A-MEXP-2196) (Mitchell et al., 2012) was used to detect the set of genes differentially expressed between the resistant population of Vallée du Kou and a susceptible laboratory colony Ngoussou. Each array contains 60 mer probes designed from all 13,000 transcripts of the Ensembl P3.5 *A. gambiae* genome annotation, plus additional probes for the detoxification genes from a previous microarray design, the ‘detox chip’, used previously to explore metabolic resistance in *A. gambiae* (David et al., 2005).

RNA was extracted from three batches of ten females 3 day old *A. gambiae* s.s. from a F_1_ sample from the VK6 population (nonexposed to insecticide but known to be resistant to multiple insecticides from bioassays results of VK) and from the Ngoussou strain which is fully susceptible to pyrethroids, DDT, carbamates and organophosphate with 100% mortality observed 24 h after 1 h exposure. RNA was isolated using the Picopure RNA isolation kit (Arcturus). The quantity and quality of extracted RNA were assessed using NanoDrop ND1000 spectrophotometer (Thermo Fisher) and Bioanalyzer (Agilent, Santa Clara, CA, USA) respectively. Complementary RNA (cRNA) of each sample was amplified using the Agilent Quick Amp labeling Kit (two-color) following the manufacturer's protocol. cRNA from the VK6 samples were labeled with cy3 dye while the cRNA from the susceptible strain Ngoussou was labeled with the cy5 dye. cRNA quantity and quality were checked before labeling using the NanoDrop and Bioanalyzer. Labeled cRNAs were hybridized to the arrays for 17 h at 65 °C according to the manufacturer's protocol. Five hybridizations between cRNA from VK and Ngoussou were carried out by swapping the biological replicates (Fig. S6).

Microarray data were analyzed using Genespring GX 11.0 software. In order to identify differentially expressed genes, a cut-off of 2-fold-change and a statistical significance of P < 0.05 and P < 0.01 were applied. The P values were generated from a *t*-test against zero using the data from the five hybridizations (Fig. S6) after a multiple testing correction using the Benjamin–Hochberg test. Enrichment analysis was carried out using the Blast2Go software (Conesa et al., 2005; Gotz et al., 2008) to detect the major Gene Ontology (GO) terms over-represented among the set of probes up or under-transcribed in the VK population in comparison to the entire microarray chip using a Fisher's test for statistical significance. The microarray data from this study were submitted to Array Express, accession number: E-MTAB-1083.

### 5.6 Validation of candidate genes using quantitative reverse transcriptase PCR

Nine of the differentially expressed genes identified from the microarray analysis were further assessed by qRT-PCR to validate their expression pattern (gene names and primer sequences are given in Table S10). One microgram of total RNA from each of the three biological replicates for VK and Ngoussou was used as template for cDNA synthesis using Superscript III (Invitrogen) with oligo-dT20 and RNase H (New England Biolabs), according to the manufacturer's instructions. A serial dilution of cDNA was used to establish standard curves for each gene in order to assess PCR efficiency and quantitative differences between samples. The qPCR amplification was performed using a MX 3005 real-time PCR system (Agilent, Santa Clara, CA, USA) with Brilliant III Ultra-Fast SYBR® Green QPCR Master Mix (Agilent, Santa Clara, CA, USA). 10 ng of cDNA from each sample was used as template in a 3-step program involving a denaturation at 95 °C for 3 min followed by 40 cycles of 10 s at 95 °C and 10 s at 60 °C and a last step of 1 min at 95 °C, 30 s at 55 °C and 95 °C at 30 s. The relative expression and fold-change of each target gene in VK relative to Ngoussou was calculated according to the 2^− ΔΔCT^ method incorporating PCR efficiency (Schmittgen and Livak, 2008) after normalization with the housekeeping genes *RSP7* ribosomal protein S7 (AGAP010592) and the GDPH (glucose dehydrogenase phosphate) (AGAP000651).

## Figures and Tables

**Table 1:** The top 50 probes the most over-transcribed in the Vallée du Kou *A. gambiae* M form population compared to the Ngoussou susceptible strain.

Probe name	Gene name	Corrected P-value	Absolute FC	Function
CUST_2693_PI422575199	AGAP007160-RB	6.22E − 04	77.1967	Alpha crystallin–small heat-shock protein
CUST_2694_PI422575199	AGAP007160-RC	5.37E − 04	75.60545	Alpha crystallin–small heat-shock protein
CUST_2692_PI422575199	AGAP007160-RA	9.12E − 04	74.3092	Alpha crystallin–small heat-shock protein
CUST_4851_PI422575199	AGAP002878-RA	0.0010	73.0915	Cysteine-type endopeptidase inhibitor activity
CUST_2394_PI422575199	AGAP006879-RA	0.0013	26.39016	ATP synthase E chain.
DETOX_487_PI422610884	CYP6Z2	0.0018	20.53136	Cytochrome P450
CUST_9916_PI422575199	AGAP011318-RA	0.0031	18.17117	Asparaginase
CUST_6539_PI422575199	AGAP012982-RA	8.92E − 04	16.65581	Putative rhodopsin receptor deltamethrin resistance-associated *NYD-OP3* in *Culex pipiens pallens*
DETOX_66_PI422610884	Aldehyde_oxidase	0.0061	16.55706	Aldehyde oxidase
CUST_1987_PI422575199	AGAP006504-RA	0.0022	15.49114	Hypothetical salivary protein SG2A
CUST_1836_PI422575199	AGAP006365-RA	0.0069	14.33992	ss-DNA binding protein 12RNP2
CUST_6404_PI422575199	AGAP004033-RA	0.0015	14.10266	Hypothetical protein
CUST_1777_PI422575199	AGAP006263-RA	9.24E − 04	13.52441	Arrestin, Arr2-like
CUST_1475_PI422575199	AGAP005996-RA	9.71E − 04	12.37302	Cuticular protein 21, RR-1 family CPR21
CUST_5632_PI422575199	AGAP003444-RA	0.0010	11.92223	Putative 13.4 kDa salivary protein
DETOX_489_PI422610884	CYP6Z2	0.0020	11.06083	Cytochrome P450
CUST_10688_PI422575199	AGAP012129-RA	0.0070	10.90494	Dimethyladenosine transferase
CUST_1367_PI422575199	AGAP005901-RA	0.0016	10.63078	SARM1; Sterile alpha and TIR motif-containing protein, putative
CUST_12342_PI422575199	AGAP009110-RA	5.37E − 04	10.52897	Conserved hypothetical protein
CUST_13058_PI422575199	AGAP009828-RA	0.0021	10.41671	Chymotrypsin 1
DETOX_461_PI422610884	CYP6P3	0.0052	9.987807	Cytochrome P450
CUST_12441_PI422575199	AGAP009212-RA	0.0036	9.820358	Serpin 6 protein
CUST_3330_PI422575199	AGAP006741-RA	9.71E − 04	9.792675	Conserved hypothetical protein
CUST_2964_PI422575199	AGAP007403-RA	0.0018	9.442213	No description
CUST_11795_PI422575199	AGAP008533-RA	0.0018	8.799952	Cyclin-dependent kinase 5 activator
CUST_8859_PI422575199	AGAP000461-RB	9.71E − 04	8.625973	Tenascin isoform g
CUST_10735_PI422575199	AGAP012179-RA	7.68E − 04	8.576783	Calbindin 53e calcium ion binding
CUST_2696_PI422575199	AGAP007161-RA	0.0011	8.46717	(response to heat) Alpha crystallin/heat shock protein
CUST_7424_PI422575199	AGAP001610-RA	0.0055	8.436959	Alpha-kinase family
CUST_3716_PI422575199	AGAP002089-RA	0.0019	8.329749	NPR3 nitrogen permease regulator of amino acid transport activity
CUST_7821_PI422575199	AGAP000646-RA	0.0015	8.208787	Alpha trans-inducing protein (alpha-TIF)
CUST_13511_PI422575199	AGAP010286-RA	9.71E − 04	8.107342	Isoform g; chromatin structure and dynamics
CUST_2186_PI422575199	AGAP006678-RA	0.0027	8.010792	No description
CUST_12137_PI422575199	AGAP008903-RA	4.05E − 05	7.86584	Leukocyte receptor [*Culex quinquefasciatus*]
DETOX_462_PI422610884	CYP6P3	0.0024	7.741744	Cytochrome P450
CUST_5633_PI422575199	AGAP003442-RA	7.37E − 04	7.734673	kDa salivary protein
CUST_11285_PI422575199	AGAP007990-RA	8.90E − 04	7.692011	Glucosyl glucuronosyl transferases
CUST_14062_PI422575199	AGAP002789-RA	0.0017	7.496881	Protein unc-13-like protein
DETOX_460_PI422610884	CYP6P3	0.0017	7.36139	Cytochrome P450
CUST_1553_PI422575199	AGAP006068-RB	9.96E − 04	7.348426	No description
CUST_3219_PI422575199	AGAP007657-RA	0.0088	7.288038	Isoform e; signal transduction mechanisms
CUST_12023_PI422575199	AGAP008782-RA	0.0012	7.245967	kDa salivary protein
CUST_308_PI422575199	AGAP004949-RA	0.0011	7.236758	No description
CUST_12435_PI422575199	AGAP009206-RA	0.0016	7.233563	GTP cyclohydrolase I feedback regulatory protein (GFRP)
CUST_8191_PI422575199	AGAP012956-RB	6.22E − 04	7.205309	No description
CUST_2641_PI422575199	AGAP007110-RA	0.0017	7.022706	No description
CUST_6548_PI422575199	AGAP013149-RA	0.0035	7.011612	Rhodopsin receptor 1
DETOX_488_PI422610884	CYP6Z2	0.0098	6.978962	Cytochrome P450
CUST_4468_PI422575199	AGAP013226-RA	0.0099	6.936893	No description
CUST_3988_PI422575199	AGAP002272-RB	5.37E − 04	6.723799	Ankyrin unc44

**Table 2:** The top 30 probes the most under-transcribed in the Vallée du Kou *A. gambiae* M form population compared to the Ngoussou susceptible strain.

Probe name	Gene name	Corrected P-value	Absolute FC	Function
CUST_6937_PI422575199	AGAP013005-RA	0.006354	31.33667	No description
CUST_6058_PI422575199	AGAP003777-RA	6.56E − 04	18.21914	No description
CUST_1289_PI422575199	AGAP005822-RA	0.003885	17.87833	Salivary protein
CUST_6303_PI422575199	AGAP003939-RC	0.001125	15.61139	CG14168 CG14168-PA
CUST_5506_PI422575199	AGAP003354-RA	5.37E − 04	15.10638	Venom allergen 5
CUST_2294_PI422575199	AGAP006782-RA	0.003609	14.07909	No description
CUST_1963_PI422575199	AGAP006480-RB	0.009903	13.98927	No description
CUST_2569_PI422575199	AGAP007043-RA	0.003609	11.93225	Urokinase-type plasminogen activator
CUST_681_PI422575199	AGAP005260-RA	0.002226	11.82989	Thymidylate kinase
CUST_11254_PI422575199	AGAP007959-RA	0.001065	11.82861	No description
CUST_5354_PI422575199	AGAP003251-RA	0.009034	11.71185	CLIPB1 protein
CUST_6623_PI422575199	AGAP004161-RA	6.22E − 04	11.12085	Isoform i
CUST_2570_PI422575199	AGAP007043-RB	0.001709	11.04052	Urokinase-type plasminogen activator
CUST_5154_PI422575199	AGAP003087-RA	0.00289	10.57497	No description
CUST_13257_PI422575199	AGAP010032-RA	6.22E − 04	10.43863	No description
CUST_5977_PI422575199	AGAP003692-RA	0.002895	10.11468	Alanyl aminopeptidase
CUST_4175_PI422575199	AGAP013481-RA	0.001121	10.02706	Conserved hypothetical protein [*Culex quinquefasciatus*]
CUST_10753_PI422575199	AGAP012197-RA	0.004419	9.860712	Histone
CUST_9966_PI422575199	AGAP011368-RA	0.004491	9.717456	Odorant-binding protein
CUST_324_PI422575199	AGAP004963-RA	0.002356	9.463758	No description
DETOX_576_PI422610884	GSTD1_5	0.001125	9.320538	GST
CUST_11713_PI422575199	AGAP008449-RA	0.001682	9.236979	Cuticle protein
CUST_4170_PI422575199	AGAP013481-RC	0.001159	9.024582	Hypothetical protein AaeL_AAEL002776 [*Aedes aegypti*]
DETOX_574_PI422610884	GSTD1_5	5.37E − 04	8.88221	GST
DETOX_812_PI422610884	TPX5	0.00186	8.838707	TPX
DETOX_623_PI422610884	GSTE4	0.002105	8.656731	GST
CUST_4174_PI422575199	AGAP013481-RG	7.84E − 04	8.569392	Hypothetical protein AaeL_AAEL002776 [*A. aegypti*]
CUST_10758_PI422575199	AGAP012202-RA	0.003722	8.315007	No description
CUST_11588_PI422575199	AGAP008311-RA	7.37E − 04	8.105989	*Drosophila melanogaster* CG14022
DETOX_575_PI422610884	GSTD1_5	0.004114	8.097907	GST
CUST_12969_PI422575199	AGAP009751-RA	0.001444	7.952107	Angiotensin-converting enzyme 2 (AGAP009751-PA)
CUST_4172_PI422575199	AGAP013481-RF	0.002015	7.8076	Hypothetical protein AaeL_AAEL002776 [*A. aegypti*]
CUST_12113_PI422575199	AGAP008879-RB	0.003038	7.678863	GM17938 [*Drosophila sechellia*]
CUST_8594_PI422575199	AGAP000260-RC	0.004567	7.644038	ATP synthase subunit mitochondrial
CUST_2918_PI422575199	AGAP007365-RA	0.001698	7.579763	Maternal protein exuperantia

**Table 3:**
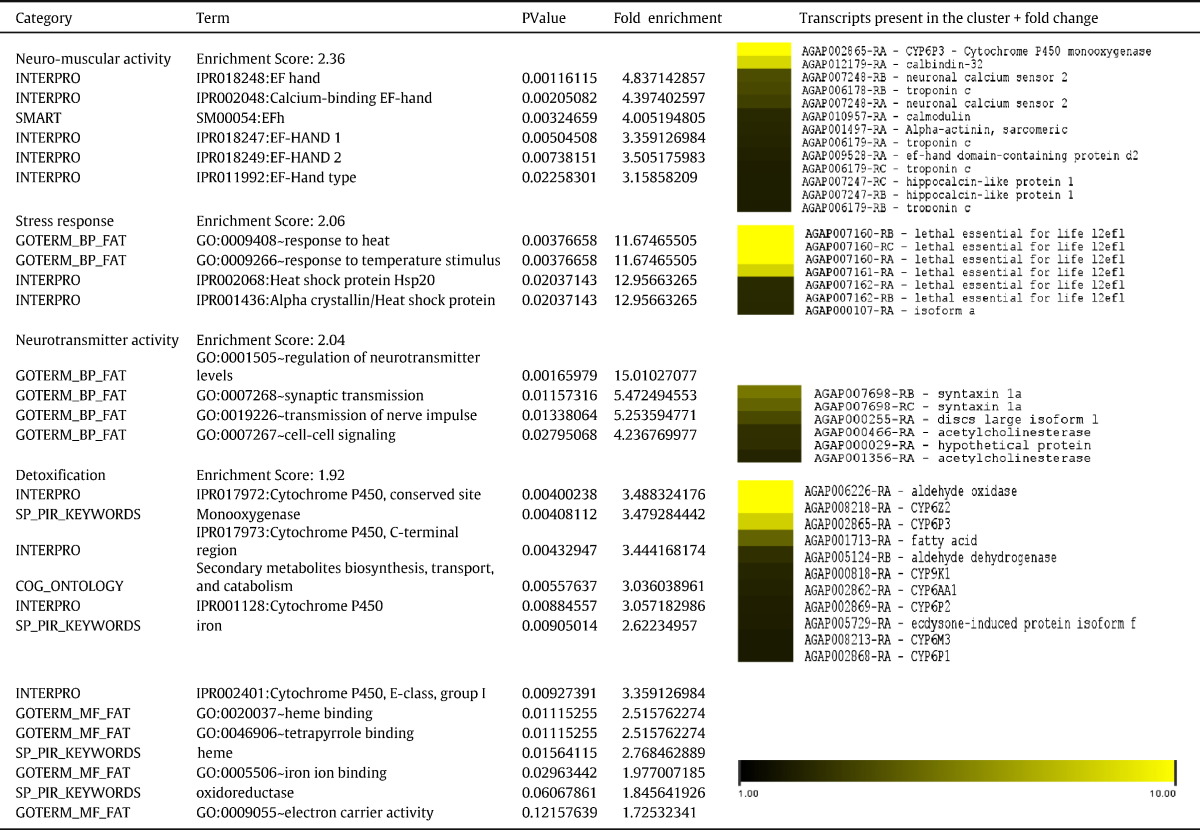
Enrichment profile of the VK over-transcribed set of probes using the DAVID functional tool.

## References

[bb0045] Chandre F. (1999). Status of pyrethroid resistance in *Anopheles gambiae* sensu lato. Bull. World Health Organ..

[bb0195] Conesa A., Gotz S., Garcia-Gomez J.M., Terol J., Talon M., Robles M. (2005). Blast2GO: a universal tool for annotation, visualization and analysis in functional genomics research. Bioinformatics.

[bb0015] Dabire K.R. (2008). Dynamics of multiple insecticide resistance in the malaria vector *Anopheles gambiae* in a rice growing area in South-Western Burkina Faso. Malar. J..

[bb0005] Dabiré K.R. (2012). Trends in insecticide resistance in natural populations of malaria vectors in Burkina Faso, West Africa: 10 years' surveys. Pest Eng..

[bb0150] David J.P. (2005). The *Anopheles gambiae* detoxification chip: a highly specific microarray to study metabolic-based insecticide resistance in malaria vectors. Proc. Natl. Acad. Sci. U. S. A..

[bb0010] Diabate A. (2002). The role of agricultural use of insecticides in resistance to pyrethroids in *Anopheles gambiae* s.l. in Burkina Faso. Am. J. Trop. Med. Hyg..

[bb0050] Diabate A. (2002). First report of the kdr mutation in *Anopheles gambiae* M form from Burkina Faso, West Africa. Parassitologia.

[bb0110] Djegbe I. (2011). Dynamics of insecticide resistance in malaria vectors in Benin: first evidence of the presence of L1014S *kdr* mutation in *Anopheles gambiae* from West Africa. Malar. J..

[bb0145] Djogbenou L., Labbe P., Chandre F., Pasteur N., Weill M. (2009). Ace-1 duplication in *Anopheles gambiae*: a challenge for malaria control. Malar. J..

[bb0055] Djouaka R.F. (2008). Expression of the cytochrome P450s, *CYP6P3* and *CYP6M2* are significantly elevated in multiple pyrethroid resistant populations of *Anopheles gambiae* s.s. from Southern Benin and Nigeria. BMC Genomics.

[bb0100] Donnelly M.J., Corbel V., Weetman D., Wilding C.S., Williamson M.S., Black W.C.T. (2009). Does *kdr* genotype predict insecticide-resistance phenotype in mosquitoes?. Trends Parasitol..

[bb0035] Du W. (2005). Independent mutations in the *Rdl* locus confer dieldrin resistance to *Anopheles gambiae* and *An. arabiensis*. Insect Mol. Biol..

[bb0075] Giraudo M. (2011). Effects of hormone agonists on Sf9 cells, proliferation and cell cycle arrest. PLoS One.

[bb0200] Gotz S. (2008). High-throughput functional annotation and data mining with the Blast2GO suite. Nucleic Acids Res..

[bb0135] Hamon J., Garrett-Jones C. (1963). Resistance to insecticides in the major malaria vectors and its operational importance. Bull. World Health Organ..

[bb0115] Hamon J., Sales S., Venard P., Coz J., Brengues J. (1968). The presence in southwest Upper Volta of populations of *Anopheles funestus* Giles resistant to dieldrin. Med. Trop. (Mars).

[bb0040] Hemingway J., Ranson H. (2000). Insecticide resistance in insect vectors of human disease. Annu. Rev. Entomol..

[bb0070] Hu X. (2007). Cloning and characterization of *NYD-OP7*, a novel deltamethrin resistance associated gene from *Culex pipiens pallens*. Pestic. Biochem. Physiol..

[bb0080] Huang da W., Sherman B.T., Lempicki R.A. (2009). Systematic and integrative analysis of large gene lists using DAVID bioinformatics resources. Nat. Protoc..

[bb0165] King C.D., Rios G.R., Green M.D., Tephly T.R. (2000). UDP-glucuronosyltransferases. Curr. Drug Metab..

[bb0175] Livak K.J. (1984). Organization and mapping of a sequence on the *Drosophila melanogaster* X and Y chromosomes that is transcribed during spermatogenesis. Genetics.

[bb0020] Martinez-Torres D. (1998). Molecular characterization of pyrethroid knockdown resistance (*kdr*) in the major malaria vector *Anopheles gambiae* s.s. Insect Mol. Biol..

[bb0190] Mitchell S.N. (2012). Identification and validation of a gene causing cross-resistance between insecticide classes in *Anopheles gambiae* from Ghana. Proc. Natl. Acad. Sci. U. S. A..

[bb0060] Muller P. (2008). Field-caught permethrin-resistant *Anopheles gambiae* overexpress *CYP6P3*, a P450 that metabolises pyrethroids. PLoS Genet..

[bb0160] Muller P. (2008). Pyrethroid tolerance is associated with elevated expression of antioxidants and agricultural practice in *Anopheles arabiensis* sampled from an area of cotton fields in Northern Cameroon. Mol. Ecol..

[bb0095] N'Guessan R., Corbel V., Akogbeto M., Rowland M. (2007). Reduced efficacy of insecticide-treated nets and indoor residual spraying for malaria control in pyrethroid resistance area, Benin. Emerg. Infect. Dis..

[bb0170] Pedra J.H., McIntyre L.M., Scharf M.E., Pittendrigh B.R. (2004). Genome-wide transcription profile of field- and laboratory-selected dichlorodiphenyltrichloroethane (DDT)-resistant *Drosophila*. Proc. Natl. Acad. Sci. U. S. A..

[bb0085] Ramphul U., Boase T., Bass C., Okedi L.M., Donnelly M.J., Muller P. (2009). Insecticide resistance and its association with target-site mutations in natural populations of *Anopheles gambiae* from eastern Uganda. Trans. R. Soc. Trop. Med. Hyg..

[bb0025] Ranson H., Jensen B., Vulule J.M., Wang X., Hemingway J., Collins F.H. (2000). Identification of a point mutation in the voltage-gated sodium channel gene of Kenyan *Anopheles gambiae* associated with resistance to DDT and pyrethroids. Insect Mol. Biol..

[bb0090] Ranson H. (2009). Insecticide resistance in *Anopheles gambiae*: data from the first year of a multi-country study highlight the extent of the problem. Malar. J..

[bb0105] Reimer L. (2008). Relationship between kdr mutation and resistance to pyrethroid and DDT insecticides in natural populations of *Anopheles gambiae*. J. Med. Entomol..

[bb0125] Rowland M. (1991). Activity and mating competitiveness of gamma HCH/dieldrin resistant and susceptible male and virgin female *Anopheles gambiae* and *An. stephensi* mosquitoes, with assessment of an insecticide-rotation strategy. Med. Vet. Entomol..

[bb0130] Rowland M. (1991). Behaviour and fitness of gamma HCH/dieldrin resistant and susceptible female *Anopheles gambiae* and *An.stephensi* mosquitoes in the absence of insecticide. Med. Vet. Entomol..

[bb0180] Santolamazza F., Mancini E., Simard F., Qi Y., Tu Z., della Torre A. (2008). Insertion polymorphisms of SINE200 retrotransposons within speciation islands of *Anopheles gambiae* molecular forms. Malar. J..

[bb0205] Schmittgen T.D., Livak K.J. (2008). Analyzing real-time PCR data by the comparative C–T method. Nat. Protoc..

[bb0140] Tantely M.L. (2010). Insecticide resistance in *Culex pipiens quinquefasciatus* and *Aedes albopictus* mosquitoes from La Reunion Island. Insect Biochem. Mol. Biol..

[bb0155] Vontas J. (2005). Gene expression in insecticide resistant and susceptible *Anopheles gambiae* strains constitutively or after insecticide exposure. Insect Mol. Biol..

[bb0030] Weill M. (2004). The unique mutation in ace-1 giving high insecticide resistance is easily detectable in mosquito vectors. Insect Mol. Biol..

[bb0065] WHO (1998). Test Procedures for Insecticide Resistance Monitoring in Malaria Vectors, Bio-efficacy and Persistence of Insecticides on Treated Surfaces.

[bb0185] Wondji C.S. (2007). Mapping a quantitative trait locus conferring pyrethroid resistance in the African malaria vector *Anopheles funestus*. BMC Genomics.

[bb0120] Wondji C.S., Dabire R.K., Tukur Z., Irving H., Djouaka R., Morgan J.C. (2011). Identification and distribution of a GABA receptor mutation conferring dieldrin resistance in the malaria vector *Anopheles funestus* in Africa. Insect Biochem. Mol. Biol..

