# Supplementary figures and images for: Dissecting the mechanisms responsible for the multiple insecticide resistance phenotype in Anopheles gambiae s.s., M form, from Vallée du Kou, Burkina Faso
Source: Gene. 2013 Apr 25;519(1):98–106. doi: 10.1016/j.gene.2013.01.036 (PMC3611593; doi:10.1016/j.gene.2013.01.036)

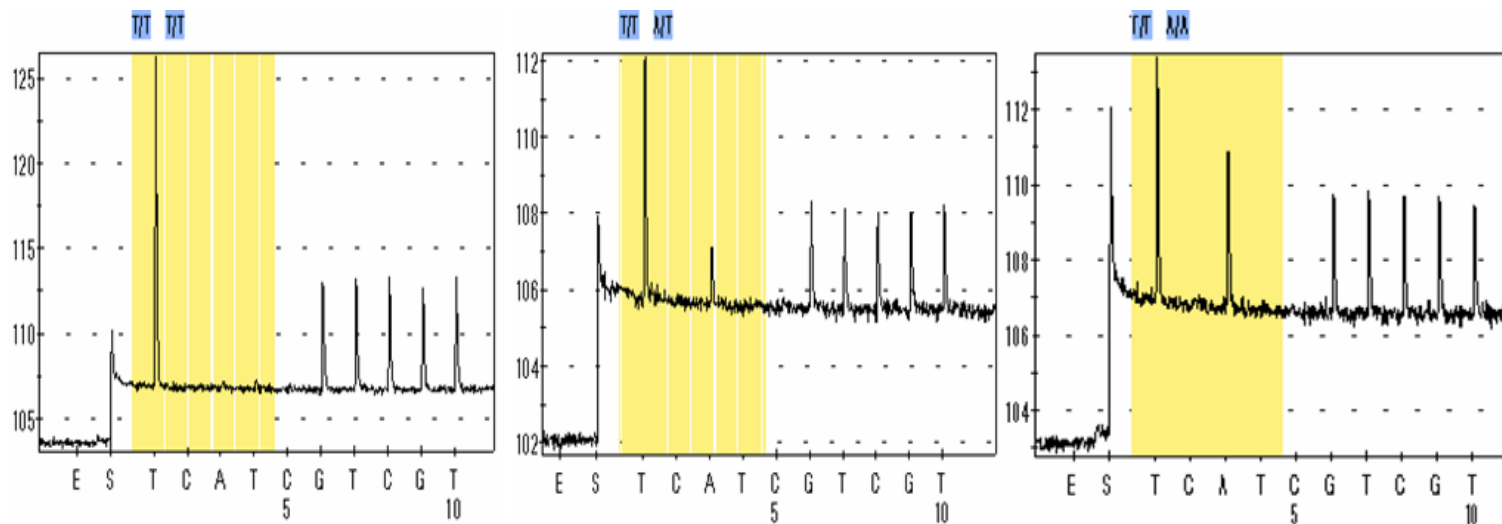

Figure S1

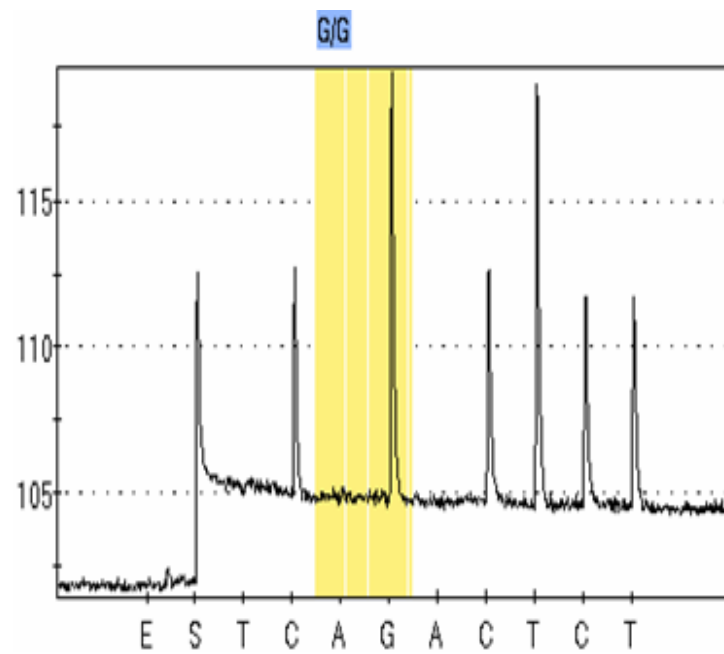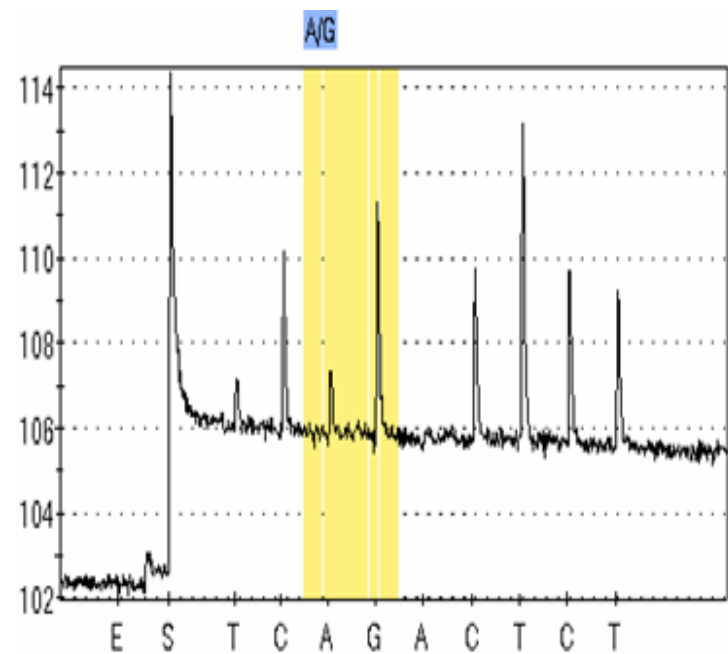

Figure S2

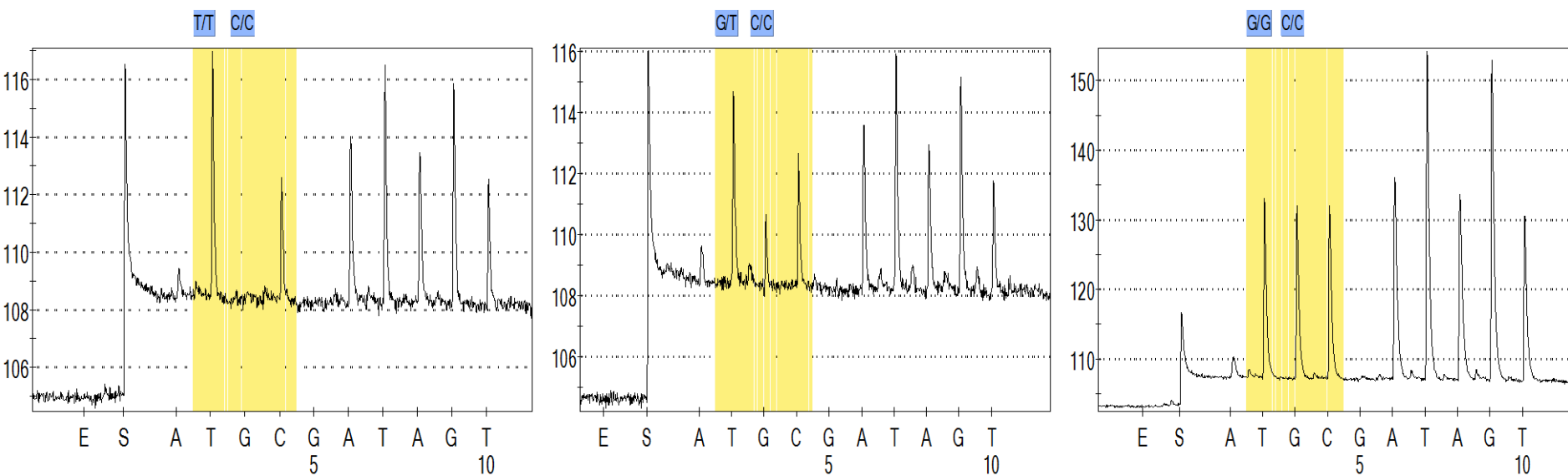

Figure S3

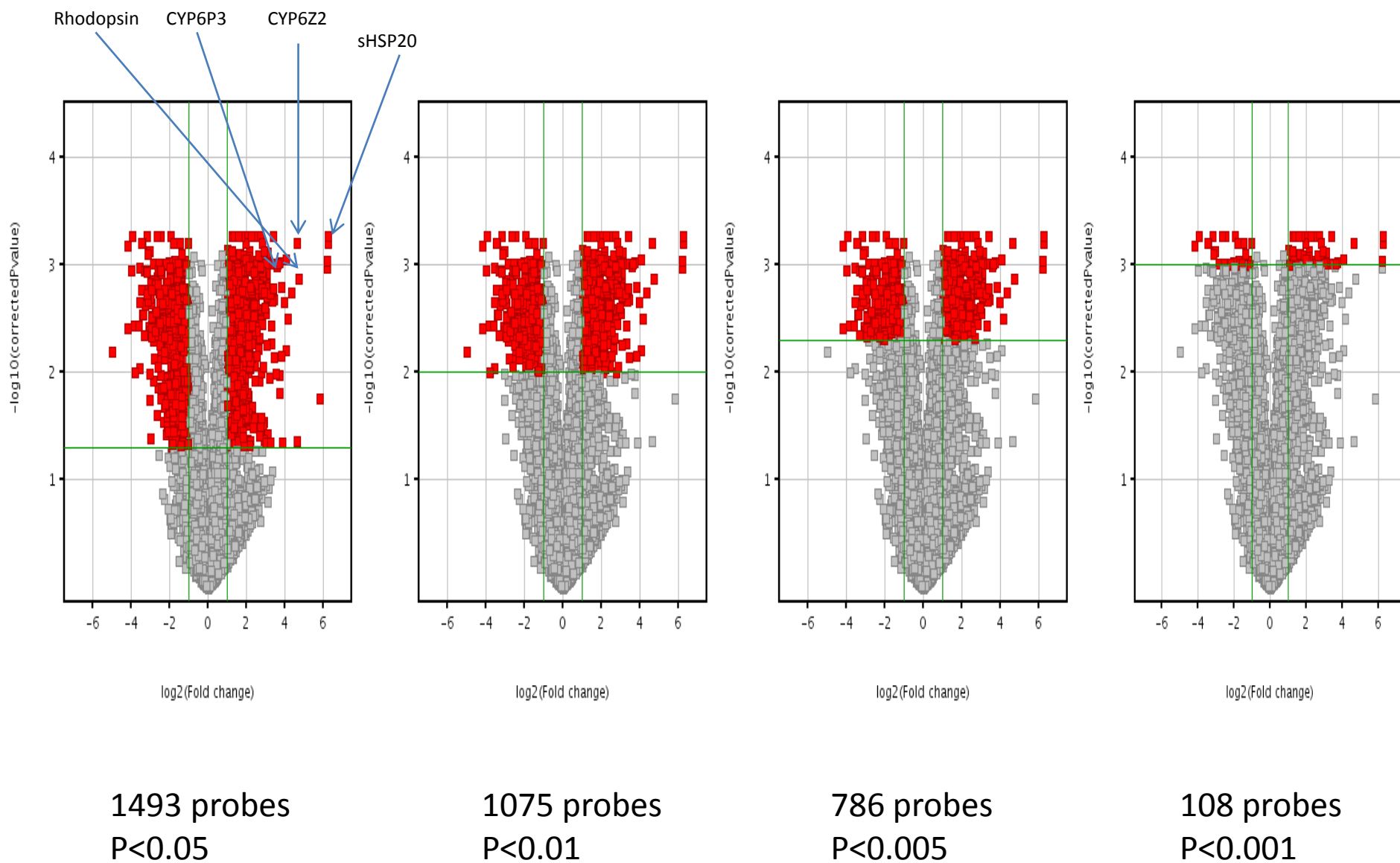

Figure S4

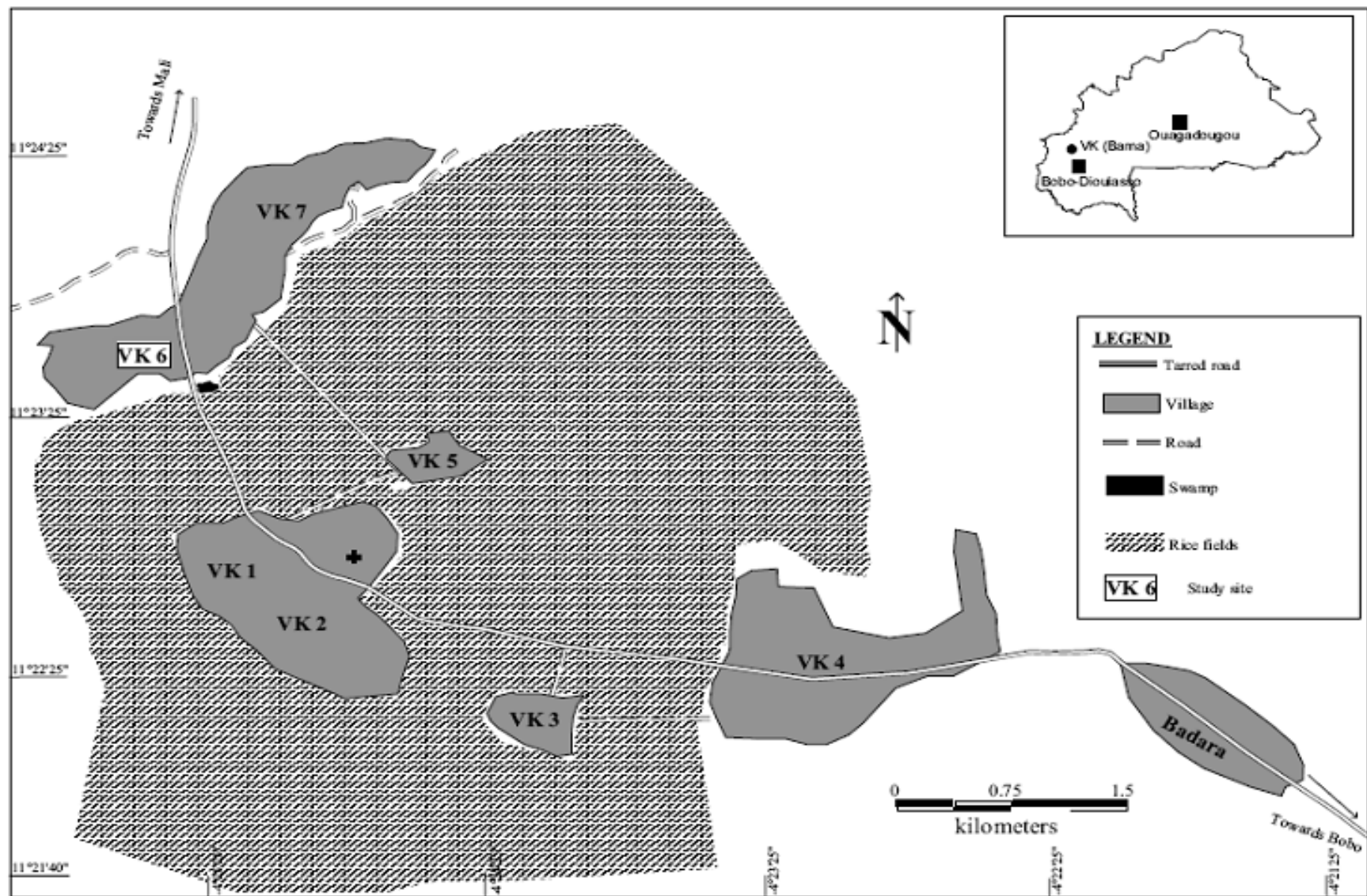

Figure S5

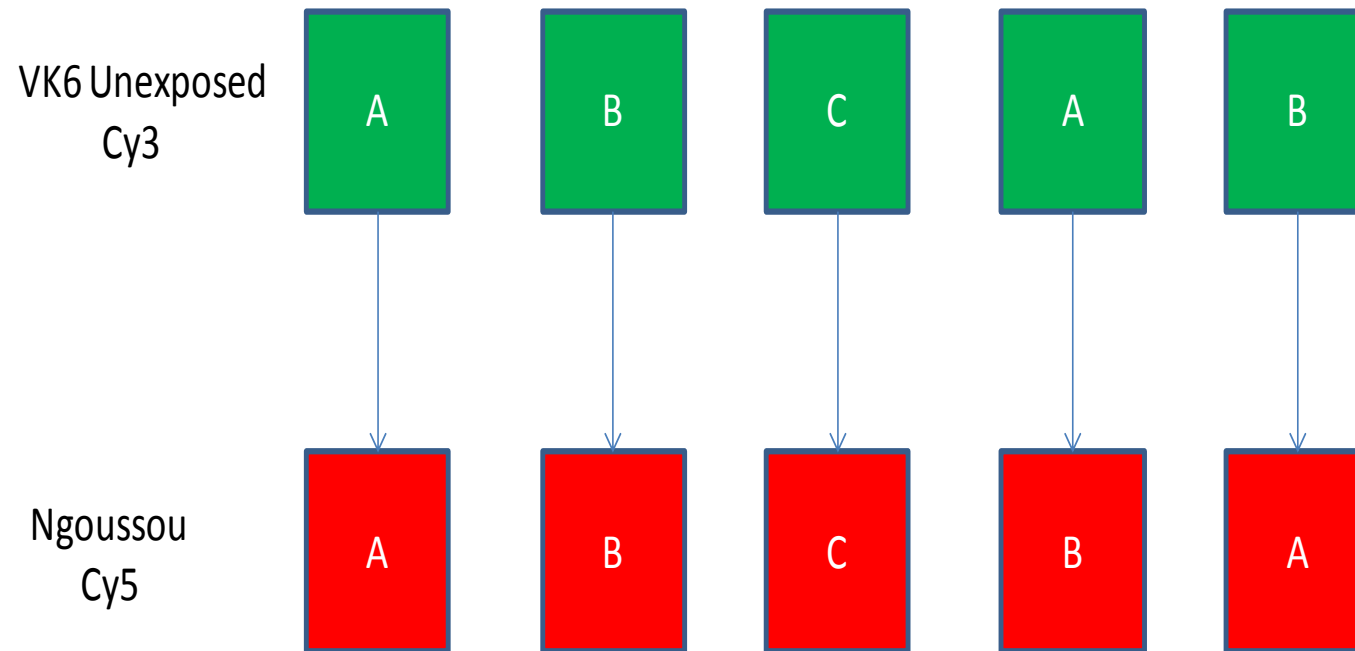

Figure S6

Supplement: Supplementary file 1 — Supplementary Table S5. [file mmc1.pdf]
