# Supplementary material for: Dissecting the mechanisms responsible for the multiple insecticide resistance phenotype in Anopheles gambiae s.s., M form, from Vallée du Kou, Burkina Faso
Source: Gene. 2013 Apr 25;519(1):98–106. doi: 10.1016/j.gene.2013.01.036 (PMC3611593; doi:10.1016/j.gene.2013.01.036)
Supplement: Supplementary file 2 — Supplementary Table S8. [file mmc2.docx]

**Table S1:** Bioassay sample size and percentage mortality by village

|  | **VK1** | | **VK2** | | **VK3** | | **VK6** | | **VK7** | | **Total** | |
| --- | --- | --- | --- | --- | --- | --- | --- | --- | --- | --- | --- | --- |
| **Insecticide** | **n** | **% mortality** | **n** | **% mortality** | **n** | **% mortality** | **n** | **% mortality** | **n** | **% mortality** | **n** | **% mortality** |
| 0.75%Permethrin | 99 | 1.01 | 98 | 4.1 | 99 | 8.08 | 99 | 3.0 | 107 | 0.93 | 502 | 3.4 |
| 4% DDT | 76 | 10.5 | 105 | 6.7 | 100 | 14 | 99 | 9.1 | / | / | 380 | 10 |
| 0.01% Bendiocarb | 93 | 94.1 | 101 | 81.2 | 101 | 91.1 | 103 | 93.2 | 106 | 96.2 | 504 | 91.1 |
| 5% Malathion | 100 | 100 | 100 | 100 | / | / | 107 | 100 | 47 | 100 | 354 | 100 |
| 4% Dieldrin | 108 | 9.3 | 101 | 4.9 | / | / | 95 | 3.2 | / | / | 304 | 5.9 |
| 0.05% Deltamethrin | / | / | 43 | 37.2 | / | / | 106 | 18.8 | / | / | 149 | 24.8 |
| 0.05% Lambda-cyhalothrin | / | / | / | / | / | / | 109 | 7.3 | / | / | 109 | 7.3 |

**Table S2:** *Kdr* Frequencies according to insecticide exposure and phenotype

| **Insecticide** | **Phenotype** | **n** | **Genotype (frequency)*** | | |
| --- | --- | --- | --- | --- | --- |
|  |  |  | **T/T (homozygote)** | **A/T (heterozygote)** | **A/A (wild type)** |
| **Permethrin** | Resistant | 24 | 14 (0.58) | 10 (0.42) | 0 |
|  | Susceptible | 14 | 3 (0.21) | 6 (0.43) | 5 (0.36) |
| **DDT** | Resistant | 23 | 11 (0.48) | 12 (0.52) | 0 |
|  | Susceptible | 21 | 4 (0.19) | 13 (0.62) | 4 (0.19) |
| * Resistant vs Susceptible significant, Permethrin: χ^2^ = 101, P = 0.000 DDT: χ^2^ = 64.9, *P* = 0.000 | | | | | |

**Table S3:** *Ace1^R^* genotype Frequencies according to insecticide exposure and phenotype

| **Insecticide** | **Phenotype** | **n** | **Genotype (frequency)*** | |
| --- | --- | --- | --- | --- |
|  |  |  | **G/G (wild type)** | **A/G (heterozygote)** |
| **Bendiocarb** | Resistant | 35 | 31 (0.89) | 4 (0.11) |
|  | Susceptible | 24 | 24 (1) | 0 |
| * No A/A homozygote genotypes were found | | | | |

**Table S4:** *RDL* Frequencies according to insecticide exposure and phenotype

| **Insecticide** | **Phenotype** | **n** | **Genotype (frequency)*** | | |
| --- | --- | --- | --- | --- | --- |
|  |  |  | **T/T (homozygote)** | **G/T (heterozygote)** | **G/G (wild type)** |
| **Dieldrin** | Resistant | 36 | 34 (0.94) | 2(0.06) | 0 |
|  | Susceptible | 40 | 4 (0.1) | 24 (0.6) | 12 (0.3) |
| * Resistant vs Susceptible significant: χ^2^ = 257, *P* = 0.000 | | | | | |

**Table S5**: List of probes differentially expressed between the resistant VK population and the susceptible Ngoussou strain (attached separately).

**Table S6**: Detoxification (or resistance-associated) genes over-transcribed in the Vallée du Kou *An. gambiae* M form population compared to the Ngoussou susceptible strain

| **Probe name** | **Corrected p-value** | **FC**  **Absolute** | **Log2 FC** | **Gene name** |  |  |
| --- | --- | --- | --- | --- | --- | --- |
| DETOX_487_PI422610884 | 0.001813 | 20.53136 | 4.359758 | CYP6Z2 |  |  |
| DETOX_66_PI422610884 | 0.006195 | 16.55706 | 4.049375 | Aldehyde_oxidase | | |
| DETOX_489_PI422610884 | 0.002085 | 11.06083 | 3.467388 | CYP6Z2 |  |  |
| DETOX_461_PI422610884 | 0.00528 | 9.987807 | 3.320168 | CYP6P3 |  |  |
| DETOX_462_PI422610884 | 0.002478 | 7.741744 | 2.952659 | CYP6P3 |  |  |
| DETOX_460_PI422610884 | 0.001717 | 7.36139 | 2.879978 | CYP6P3 |  |  |
| DETOX_488_PI422610884 | 0.009804 | 6.978962 | 2.803012 | CYP6Z2 |  |  |
| DETOX_706_PI422610884 | 0.001485 | 3.480863 | 1.799445 | PX13A |  |  |
| DETOX_34_PI422610884 | 0.00312 | 2.736745 | 1.452461 | ACE2 |  |  |
| DETOX_550_PI422610884 | 0.00172 | 2.655742 | 1.409115 | GPX3 |  |  |
| DETOX_672_PI422610884 | 0.001382 | 2.580334 | 1.367558 | GSTZ1 |  |  |
| DETOX_31_PI422610884 | 9.96E-04 | 2.476101 | 1.30807 | ACE1 |  |  |
| DETOX_410_PI422610884 | 0.001687 | 2.40752 | 1.267548 | CYP6AA1 |  |  |
| DETOX_425_PI422610884 | 0.005047 | 2.334325 | 1.223005 | CYP6AG2 |  |  |
| DETOX_411_PI422610884 | 0.003791 | 2.303939 | 1.204102 | CYP6AA1 |  |  |
| DETOX_426_PI422610884 | 0.003161 | 2.302166 | 1.202992 | CYP6AG2 |  |  |
| DETOX_32_PI422610884 | 0.005089 | 2.26426 | 1.179039 | ACE1 |  |  |
| DETOX_409_PI422610884 | 0.001485 | 2.152987 | 1.10634 | CYP6AA1 |  |  |
| DETOX_33_PI422610884 | 0.005461 | 2.147745 | 1.102823 | ACE1 |  |  |
| DETOX_443_PI422610884 | 0.008082 | 2.020629 | 1.014804 | CYP6M3 |  |  |
| DETOX_455_PI422610884 | 0.006804 | 2.002004 | 1.001445 | CYP6P1 |  |  |

**Table S7**: Detoxification (or resistance-associated) genes under-transcribed in the Vallée du Kou  *An. gambiae* M form population compared to the Ngoussou susceptible strain

| **Probe name** | **p-value** | **FC**  **Absolute** | **Log2** | **Gene name** |  |  |
| --- | --- | --- | --- | --- | --- | --- |
| DETOX_576_PI422610884 | 0.001125 | 9.320538 | 3.220413 | GSTD1-5 |  | |
| DETOX_574_PI422610884 | 5.37E-04 | 8.88221 | 3.150919 | GSTD1-5 |  | |
| DETOX_812_PI422610884 | 0.00186 | 8.838707 | 3.143835 | TPX5 |  | |
| DETOX_623_PI422610884 | 0.002105 | 8.656731 | 3.113822 | GSTE4 |  | |
| DETOX_575_PI422610884 | 0.004114 | 8.097907 | 3.017549 | GSTD1-5 |  | |
| DETOX_700_PI422610884 | 0.001529 | 6.130671 | 2.616045 | PX11 |  | |
| DETOX_620_PI422610884 | 0.001125 | 6.071878 | 2.602143 | GSTE3 |  | |
| DETOX_702_PI422610884 | 0.00426 | 5.915664 | 2.56454 | PX11 |  | |
| DETOX_622_PI422610884 | 0.001165 | 5.737644 | 2.520458 | GSTE4 |  | |
| DETOX_619_PI422610884 | 0.00186 | 5.665702 | 2.502255 | GSTE3 |  | |
| DETOX_577_PI422610884 | 0.001444 | 4.274606 | 2.095791 | GSTD1-6 |  | |
| DETOX_578_PI422610884 | 9.71E-04 | 4.216194 | 2.075941 | GSTD1-6 |  | |
| DETOX_435_PI422610884 | 0.002668 | 3.886617 | 1.958515 | CYP6AK1 |  | |
| DETOX_579_PI422610884 | 0.00474 | 3.838377 | 1.940496 | GSTD1-6 |  | |
| DETOX_774_PI422610884 | 0.00426 | 3.837997 | 1.940354 | SP11644 |  | |
| DETOX_699_PI422610884 | 0.004989 | 3.716561 | 1.893968 | PX10 |  | |
| DETOX_434_PI422610884 | 0.005135 | 3.642888 | 1.865082 | CYP6AK1 |  | |
| DETOX_643_PI422610884 | 0.0038 | 3.613722 | 1.853485 | GSTO1 |  | |
| DETOX_772_PI422610884 | 0.002644 | 3.514603 | 1.813362 | SP11644 |  | |
| DETOX_757_PI422610884 | 0.004515 | 3.3579 | 1.747559 | SOD1 |  | |
| DETOX_433_PI422610884 | 0.006226 | 3.334415 | 1.737434 | CYP6AK1 |  | |
| DETOX_835_PI422610884 | 0.003885 | 3.263361 | 1.706359 | XD18014 |  | |
| DETOX_773_PI422610884 | 0.006595 | 3.233335 | 1.693023 | SP11644 |  | |
| DETOX_758_PI422610884 | 0.004264 | 3.130855 | 1.646557 | SOD1 |  | |
| DETOX_770_PI422610884 | 0.00548 | 3.080498 | 1.623164 | SP11372 |  | |
| DETOX_528_PI422610884 | 0.002053 | 2.861401 | 1.516722 | Cytochrome c |  |  |
| DETOX_769_PI422610884 | 0.001165 | 2.800488 | 1.485678 | SP11372 |  | |
| DETOX_559_PI422610884 | 0.007474 | 2.714325 | 1.440593 | GRX2 |  | |
| DETOX_78_PI422610884 | 0.001586 | 2.52673 | 1.337272 | CAT1 |  | |
| DETOX_837_PI422610884 | 0.002356 | 2.476458 | 1.308278 | XD18014 |  | |
| DETOX_660_PI422610884 | 0.007697 | 2.422175 | 1.276303 | GSTU1 |  | |
| DETOX_658_PI422610884 | 0.008439 | 2.408856 | 1.268348 | GSTU1 |  | |
| DETOX_644_PI422610884 | 0.009599 | 2.365064 | 1.241879 | GSTO1 |  | |
| DETOX_678_PI422610884 | 0.003637 | 2.361415 | 1.239652 | NADH dehydrogenase | |  |
| DETOX_676_PI422610884 | 0.002807 | 2.114295 | 1.080177 | NADH dehydrogenase | |  |

**Table S**8 List of Gene Ontology (GO) terms enriched for the probes over-transcribed in VK (attached separately)

**Table S9:** *Kdr,* *AChE* and RDL pyrosequencing primers information.

|  | **Kdr (L1014F and L1014S)** | **G119S Ace-1** | **A296S RDL** |
| --- | --- | --- | --- |
| Forward primer | TTGTGTTCCGTGTGCTATGC | CCTGTCCGAGGACTGTCTGT | TCGTGGGTATCATTTTGGCTA |
| Biotinalated Reverse primer | AAAAACGATCTTGGTCCATGT | ACCACGATCACGTTCTCCTC | ATGACGAAGCATGTGCCTAA |
| Sequencing | TGTAGTGATAGGAAAT | TGTGGATCTTCGGCGG | CTACACCAGCACGTGT |
| Sequence to analyse | T C/T A/T GTCGTAAG | C A/G GCTTCTACTCC | T G/T G/C ATTAGGTGT |
| Product size (bp) | 154 | 165 | 167 |
| Allele | C/T//A/T | A/G | G/T |

**Table S10:** Primers used for qRT-PCR with the MX 3005 real-time PCR system

| **Genes** | **Forward Primer** | **Reverse Primer** | **Expected size (bp)** |
| --- | --- | --- | --- |
| sHSP20 (AGAP007160) | GATCTGGCACACCGGTAAAC | CCGAAGGGAATACGATGTGT | 113 |
| Rhodopsin (AGAP012982) | TGCCCTGTACCAGAAGTTCC | TTCCGCTCTCTTTCTTACGC | 120 |
| Arrestin (AGAP006263-RA) | GATGAACGAGCCGGAGTAAG | CTGTCCCCTCTCCGGATTA | 100 |
| Aldehyde oxidase | GATATTCCGGTCGACTTTCG | AACGACACGGTCATGTTGAG | 110 |
| CYP6P3 | AGCGATGCTTTGTTTTGCTT | AGCGATGCTTTGTTTTGCTT | 131 |
| CYP6Z2 | AGTTCAAGTTCCAGGCCACA | TTTAGTTCATCACAATCAGTTGC | 138 |
| RSP7 (AGAP010592) | GTGTTCGGTTCCAAGGTGAT | TCCGAGTTCATTTCCAGCTC | 98 |
| Acetylcholinesterase 1 (Ace-1) | GTTGCAGCTACCTCGAACCT | ACACCAGCAGCACGATCA | 100 |
| UDPGT(AGAP007990) | CGTGCTGACGTTCTATCTCG | ACCTTCGATTTTGTGCTGCT | 108 |
| CYP6M3 | TGGACCAGATACTGAAGGAGAGT | ACAGAGGTTCCTGCTTCGAG | 121 |
| GDPH (AGAP000651) | CTGCAAAAAGTCGATACCGC | CCTCGTACACGTACATCGTGA | 170 |
